# Supplementary material for: PplD is a de-N-acetylase of the cell wall linkage unit of streptococcal rhamnopolysaccharides
Source: Nat Commun. 2022 Feb 1;13:590. doi: 10.1038/s41467-022-28257-0 (PMC8807736; doi:10.1038/s41467-022-28257-0)
Supplement: Supplementary file 1 — Supplementary Information [file 41467_2022_28257_MOESM1_ESM.pdf]

Supplementary information for:

PplD is a de-N-acetylase of the cell wall linkage unit of streptococcal rhamnopolysaccharides

Jeffrey S. Rush[1], Prakash Parajuli[2], Alessandro Ruda[3], Jian Li[1], Amol Arunrao Pohane[2], Svetlana Zamakhaeva[2], Mohammad M. Rahman[2], Jennifer C. Chang[4], Artemis Gogos[4], Cameron W. Kenner[2], Gérard Lambeau[5], Michael J. Federle[4], Konstantin V. Korotkov[1], Göran Widmalm[3], and Natalia Korotkova[1, 2*]

[1]Department of Molecular and Cellular Biochemistry, University of Kentucky, Lexington, Kentucky, USA

[2]Department of Microbiology, Immunology and Molecular Genetics, University of Kentucky, Lexington, Kentucky, USA

[3]Department of Organic Chemistry, Arrhenius Laboratory, Stockholm University, Stockholm, Sweden

[4]Department of Medicinal Chemistry and Pharmacognosy, University of Illinois at Chicago, Chicago, Illinois, USA

[5]Université Côte d'Azur, Centre National de la Recherche Scientifique, Institut de Pharmacologie Moléculaire et Cellulaire, Valbonne Sophia Antipolis, France

*Correspondence and request for materials should be addressed to N.K. (email: nkorotkova@uky.edu)

Supplementary Table 1. ^{1}H, ^{13}C and ^{31}P NMR chemical shifts (ppm) at 50 °C of the linker region from Group A Carbohydrate and inter-residue correlations from ^{1}H,^{1}H-NOESY, ^{1}H,^{13}C-HMBC and ^{1}H,^{31}P-HMBC experiments.

**Sugar residue**	**^{1}H/^{13}C**								**^{31}P**	**Inter-residue correlation**	
	1	2	3	4	5	6	α	β	PO_4	NOE	HMBC[b]
→3)-β-L-Rha*p*-(1→	4.89 {164}[a]	4.18	3.65	3.50	3.43	1.33				H4, **GlcN**	C4, **GlcN**
	101.6	71.4	81.4	72.1	72.9	17.6					
→4)-α-D-Glc*p*N(1-*P*	5.54 {177}	2.89	3.94	3.69	3.77	~3.88			-1.35		H1, **GlcN**<br>H2, **GlcN**
	96.4	56.0	72.9	77.7	73.6	61.5					
P-6)-β-D-Mur*p*NAc-(1→	4.57 {167}	3.85	3.64	3.93	3.47	4.10, 4.20	4.37	1.41			H6a, **MurNAc**<br>H6b, **MurNAc**
	102.4	55.9	80.4	75.9	75.0	64.4	79.2	19.0			

[a] $^{1}J_{CH}$ values are given in Hertz in braces; [b] Refers to ^{1}H,^{13}C-HMBC or ^{1}H,^{31}P-HMBC experiments.

Supplementary Table 2. Binding of GFP-AtlA^{Efs} to the GAS sacculi.

	Total number of cells counted	Number of cells with GFP-AtlA^{Efs} bound to the poles	Number of cells with GFP-AtlA^{Efs} bound to the whole cell surface	Number of dividing cells	Number of dividing cells with GFP-AtlA^{Efs} bound to the septal regions
Untreated	127	34 (26%)[a]	0	41	8 (19%)[c]
Treated with mild acid	183	129 (70%)	0	70	65 (92.9%)
Treated with nitrous acid	117	117 (100%)	117 (100%)[b]	47	117 (100%)

[a] The percentage of cells with GFP-AtlA^{Efs} attached to the poles.

[b] The percentage of cells with GFP-AtlA^{Efs} attached to the whole cell surface.

[c] The percentage of dividing cells with GFP-AtlA^{Efs} attached to the septal regions.

Supplementary Table 3. Data collection and refinement statistics for PplD structure.

	PDB: 6DQ3[1]
**Data collection**	
Space group	$P2_12_12_1$
Cell dimensions	
a, *b*, *c* (Å)	42.16, 78.43, 138.07
α, β, γ (°)	90, 90, 90
	Peak
Wavelength (Å)	1.2700
Resolution (Å)	39.69–1.78 (1.83–1.78)[2]
R_{sym} or R_{merge}	0.065 (1.126)[3]
$I / \sigma I$	13.04 (1.09)
Completeness (%)	99.3 (97.5)
Redundancy	3.8 (3.3)
**Refinement**	
Resolution (Å)	39.69–1.78
No. reflections (total / free)	84025 / 4297
R_{work} / R_{free}	0.180 / 0.216
No. atoms	
Protein	3597
Ligand/ion	37
Water	296
B-factors	
Protein	35.1
Ligand/ion	39.6
Water	38.1
Wilson *B*	33.5
R.m.s deviations	
Bond lengths (Å)	0.004
Bond angles (°)	0.685

[1]One crystal was used for data collection.
[2]Values in parentheses are for highest-resolution shell.
[3]Friedel pairs are treated as separate reflections.

Supplementary Table 4. Structural homologs of GAS PplD.

PDB ID	Z score	r.m.s.d.	Number of aligned residues	Total number of residues	Sequence identity, %	Protein function	Reference
4hd5	30.3	1.4	209	316	31	*Bacillus cereus*, polysaccharide deacetylase	[1]
4v33	30.1	1.5	211	316	30	*Bacillus anthracis*, polysaccharide deacetylase-like protein	[2]
6go1	29.2	1.7	211	318	27	*Bacillus anthracis*, polysaccharide deacetylase-like protein	[3]
4wcj	26.7	2.1	208	233	24	*Ammonifex degensii*, polysaccharide deacetylase	[4]
4u10	26.3	2.7	221	264	19	*Aggregatibacter actinomycetemcomitans*, poly-β-1,6-N-acetyl-D-glucosamine N-deacetylase	NP[a]
3vus	25.8	2.3	215	263	21	*Escherichia coli*, poly-β-1,6-N-acetyl-D-glucosamine N-deacetylase	[5]
4f9d	25.7	2.5	219	592	21	*Escherichia coli*, poly-β-1,6-N-acetyl-D-glucosamine N-deacetylase	[6]
5bu6	22.6	2.5	197	264	19	*Bordetella bronchiseptica*, poly-β-1,6-N-acetyl-D-glucosamine N-deacetylase	[7]
2c1g	11.7	2.4	140	384	26	*Streptococcus pneumoniae*, peptidoglycan GlcNAc deacetylase	[8]

[a]NP – no publication.

Supplementary Table 5. Bacterial strains and plasmids.

Strain or plasmid	Description [a]	Reference
Bacteria		
GAS NZ131	M49-serotype strain	[9]
GAS 5005	M1T1-serotype strain	[10]
S. mutans Xc	Serotype *c* strain	[11]
S. equi	*S. equi* subsp. *equi* CF32 was isolated from an equine submandibular abscess.	[12]
S. agalactiae A909	Human clinical isolate. Serotype Ia strain, ST-7	ATCC
S. agalactiae COH1	Clinical isolate obtained from an infected newborn with sepsis. Serotype III strain, ST-17	[13]
S. thermophilus LMG 18311	Isolated from yogurt manufactured in the United Kingdom	ATCC
GASΔ*pgdA*	*pgdA* deletion mutant in the GAS NZ131 strain background (has a nonpolar kanamycin resistance cassette replacing *pgdA*), Kan^R	[14]
GASΔ*pplD*	*pplD* deletion mutant in the GAS NZ131 strain background (has a nonpolar kanamycin resistance cassette replacing *pplD*), Kan^R	This study
GASΔ*pplD*Δ*pgdA*	*pplD* deletion mutant in the GASΔ*pgdA* strain background (has nonpolar spectinomycin and kanamycin resistance cassettes inserted in *pplD* and *pgdA*, respectively), $Spec^R$, Kan^R	This study
GASΔ*pplD:*p*pplD*	GASΔ*pplD* is complemented with p*pplD* carrying GAS WT *pplD,* Kan^R, Cam^R	This study
GASΔ*pplD:*p*pplD-H105A*	GASΔ*pplD* is complemented with p*pplD* carrying a catalytically inactive variant of GAS *pplD*-H105A*,* Kan^R, Cam^R	This study
GASΔpplD:p*pplD*-D167N	GASΔ*pplD* is complemented with p*pplD* carrying a catalytically inactive variant of GAS *pplD*-D167N, Kan^R, Cam^R	This study
GASΔ*pplD:*p*pplD--H105A/D167N*	GASΔ*pplD* is complemented with p*pplD* carrying a catalytically inactive variant of GAS *pplD*-H105A/D167N*,* Kan^R, Cam^R	This study
GASΔ*pplD*Δ*pgdA:*p*pplD*	GASΔ*pplD*Δ*pgdA* is complemented with p*pplD* carrying GAS WT *pplD*, $Spec^R$, Kan^R, Cam^R	This study
GASΔ*gacH*	*gacH* deletion mutant in the GAS NZ131 strain background (has a nonpolar chloramphenicol resistance cassette inserted in *gacH*), Cam^R	This study
GASΔ*gacH*:p*gacH*	GASΔ*gacH* is complemented with p*gacH*_erm carrying WT *gacH*, Cam^R, Erm^R	This study
GASΔ*gacH:*p*gacH*-T530A	GASΔ*gacH* is complemented with p*gacH*-T530A *c*arrying a catalytically inactive variant of *gacH*, Cam^R, Erm^R	This study
GBSΔ*pplD*	*pplD* deletion mutant in the GBS A909 strain background (has a nonpolar spectinomycin resistance cassette replacing *pplD*), $Spec^R$	This study
GBSΔ*pplD:*p*pplD*	GBSΔ*pplD* is complemented with p*pplD* carrying GAS WT *pplD,* $Spec^R$, Cam^R	This study

SMUΔ*pplD*	*pplD* deletion mutant in the *S. mutans* Xc strain background (has a nonpolar spectinomycin resistance cassette replacing *pplD*), $Spec^R$	This study
SMUΔ*pplD*:p*pplD*	SMUΔ*pplD* is complemented with p*pplD* carrying GAS WT *pplD,* $Spec^R$, Cam^R	This study
SMUΔ*pplD*:p*pplD*-H105A	SMUΔ*pplD* is complemented with p*pplD* carrying a catalytically inactive variant of GAS *pplD*-H105A*,* $Spec^R$, Cam^R	This study
SMUΔ*pplD*:p*pplD*-D167N	SMUΔ*pplD* is complemented with p*pplD* carrying a catalytically inactive variant of GAS *pplD*-D167N, $Spec^R$, Cam^R	This study
SMUΔ*pplD*:p*pplD*-H105A/D167N	SMUΔ*pplD* is complemented with p*pplD* carrying a catalytically inactive variant of GAS *pplD*-H105A/D167N*,* $Spec^R$, Cam^R	This study
Escherichia coli		
DH5α	*E. coli* cells used for cloning	Invitrogen
Rosetta (DE3)	*E. coli* cells used for protein expression; Cam^R	Novagen
Plasmids		
pHY304	A temperature sensitive *E.coli-Streptococcus* shuttle vector, Erm^R	[15]
pHY304Δ*gacH*-NZ131	Derivative of pHY304 expressing a nonpolar chloramphenicol resistance cassette flanked with GAS NZ131 *gacH* 5' and 3' regions, Cam^R, Erm^R	This study
pUC19BXspec	Derivative of pUC19BX expressing *aadA* (a nonpolar spectinomycin) with own rbs, Amp^R	[16]
pUC19BXspec-*pplD*	Derivative of pUC19BXspec expressing *aadA* flanked with *pplD* 5' and 3' regions, Amp^R	This study
pHY304Δ*pplD*-NZ131	Derivative of pHY304 expressing a nonpolar spectinomycin resistance cassette flanked with GAS NZ131 *pplD* 5' and 3' regions, $Spec^R$, Erm^R	This study
pHY304Δ*pplD*-GBS	Derivative of pHY304 expressing a nonpolar spectinomycin resistance cassette flanked with GBS A909 *pplD* 5' and 3' regions, $Spec^R$, Erm^R	This study
pFED760	A temperature sensitive *E.coli-Streptococcus* shuttle vector, Erm^R	[17]
pOskar	Vector encoding kanamycin resistance cassette, Kan^R	[18]
pLR16T	Vector encoding spectinomycin resistance cassette, $Spec^R$	[19]
pDC123	*E. coli-streptococcus* shuttle vector, JS-3 replicon, chloramphenicol resistance cassette. Cam^R	[20]
p*pplD*	pDC123 derived plasmid expressing *pplD.* Cam^R	This study
p*pplD*-H105A	pDC123 derived plasmid expressing *pplD*-H105A, Cam^R	This study
p*pplD*-D167N	pDC123 derived plasmid expressing *pplD*-D167N, Cam^R	This study
p*pplD*-H105A/D167N	pDC123 derived plasmid expressing *pplD*-H105A/D167N, Cam^R	This study
pDCerm	pDC123 derivative with Erm (Erm of Tn916ΔE) replacing chloramphenicol resistance cassette, Erm^R	[21]

p*gacH*_erm	pDCerm derived plasmid expressing *gacH,* Erm^R	[22]
p*gacH*-T530A	p*gacH*_erm derived plasmid expressing a catalytically inactive of variant of *gacH,* Erm^R	[22]
pRSF-NT	A modified pRSF-Duet1 (Novagen) vector that allows the creation of N-terminus His-tagged proteins with a TEV protease cleavage site, Kan^R	[23]
pKV1644	pRSF-NT derived plasmid for expression of GFP-$AtlA^{Efs}$. A C-terminal cell wall-binding domain of AtlA from *E. faecalis* fused with GFP at the N-terminus. GFP has a His-tag followed by a TEV protease recognition site at the N-terminus, Kan^R	This study
pCDF-NT	A modified pCDF-Duet1 (Novagen) vector for expression of the N-terminus His-tagged proteins with a TEV protease cleavage site, Str^R	[24]
pCDF-PplD	pCDF-NT derived plasmid expressing ePplD fused with N-terminal TEV protease cleavable His-tag, Str^R	This study

[a] Antibiotic resistance markers: Erm^R, erythromycin; Kan^R, kanamycin; $Spec^R$, spectinomycin; Cam^R, chloramphenicol, Amp^R, ampicillin, Str^R, streptomycin

Supplementary Table 6. Primers

Primer	Sequence[a,b]	Genetic manipulations
JC480	CATGGAATTCCCAGATTGAAGCACCAACC	GAS *pplD* deletion with a nonpolar kanamycin resistance cassette
JC481	CATGACGCGTTACTTGCTCCTTTTTTTGATAT	
JC482	CATGACGCGTCATCAATTTTTTTATCATTCCAAA	
JC483	CATGGAATTCAACTTATTCAGTTCAAGACCTG	
JC292	GCATGACGCGTATGGCTAAAATGAGAATATCACC	
JC304	GCATGACGCGTCTAAAACAATTCATCCAGTAAAATATAA	
PplD-BglII-F	GCGTAAGATCTGTGATCATGAATCCATTCTAG	GAS *pplD* deletion with a nonpolar spectinomycin resistance cassette
PplD-SalI-R	CGCTGCGTCGACGGGTTGACAATCAAATTAGC	
PplD-BamH-F	CGTCTGGATCCCAGTATGATTGATTTCTACAAC	
PplD-XhoI-R	GCGCGCTCGAGGACGTCTCCAAGAATAACAGC	
GacHNZ-BamH-F2	CGTCTGGATCCCGTTGGTCCCGCTAAAGCAATG	GAS NZ131 *gacH* deletion with a nonpolar chloramphenicol resistance cassette
cm-gacHNZ-R1	**GTGAATTTAGGAGGC**CGTATATGATTGTTGCAAATATG	
gacHNZ-cam-F1	CAACAATCATATACG**GCCTCCTAAATTCACTTTAG**	
Cm-gacHNZ-F2	**AATATGAGATAATGC**GGTGATGAAGCATTGCTAGG	
gacHNZ-Cm-R2	CAATGCTTCATCACC**GCATTATCTCATATTATAAAAG**	
GacHNZ-XhoI-R	GCGCGCTCGAGCATAAGTCCCGCAGTTGTGC	
PplD-Smu-F	CCGCTTCTTGTGCTATAATAAG	*S. mutans pplD* deletion with a nonpolar spectinomycin resistance cassette
Spec-PplD-Smu-r1	**CTCACTATTTTGGTCGAC**CTTTAGCTCTGCAGCCTGCG	
PplD-Smu-Spec-f1	CAGGCTGCAGAGCTAAAG**GTCGACCAAAATAGTGAGGAG**	
Spec-PplD-Smu-f2	**AAAAATTATAAGGATCC**CAGCAATAGCTTATCCAGCGG	
PplD-Smu-Spec-r2	CTGGATAAGCTATTGCTG**GGATCCTTATAATTTTTTAATCTG**	
PplD-Smu-XhoI-R	GGAAGAAATATGTTTGGAAGC	
PplD-GBS-BamHI-F	CGTCTGGATCCGTATTTATCTCTGTCACAAAG	GBS *pplD* deletion with a nonpolar spectinomycin resistance cassette
Spec-PplD-GBS-R1	**CTCACTATTTTGGTCGAC**GCTAAGAAGTAATATCCTTCC	
PplD-GBS-spec-F1	GATATTACTTCTTAGC**GTCGACCAAAATAGTGAGGAG**	
Spec-PplD-GBS-F2	**AAAATTATAAGGATC**GATGATGGAAATGCCGATTTC	
PplD-GBS-spec-R2	GGCATTTCCATCATC**GATCCTTATAATTTTTTAATCTG**	

PplD-GBS-XhoI-F	GCGCGCTCGAGTTGGCAGTGATTGCTATTG	
sfGFP_BspH	GAGATCATGAGCAAAGGAGAAGAACTTTTCAC	Construction of pKV1644
gfp-0799-R	**GCCCCCAGTATT**ACCACCTCCACCTTTGTAGAGC	
gfp-0799-F	ACAAAGGTGGAGGTGGT**AATACTGGGGGCGGAAC**	
0799-Hind	CAGAAGCTTAACCAACTTTTAAAGTTTGACCAATATAAATTG	
PplD-NcoI-f	CGTGAGCCATGGAAACACCTGTCAAGATCCC	Construction of pCDF-PplD
PplD-XhoI-r2	GCGCGCTCGAGTTATGGTTCCATTGTTTGTAAAAG	
PplD-HindIII-f	CGCGCAAGCTTGTTACAATAGAGGTACTTATATC	Construction of p*pplD*
PplD-BamHI-r2	CGTCTGGATCCTTATGGTTCCATTGTTTGTAAAAG	
PplD-GAS-check-f	CTTGGACCAAGCAAGAAACAC	Verification of GASΔ*pplD*
PplD-GAS-check-r	CTTCCCATTTCGGTTAGTCC	
gacHNZ-check-F	CTGTGGATAGTTTTACTTGTC	Verification of GASΔ*gacH*
gacHNZ-check-R	CAACAGAAATAATTGTTCCC	
PplD-SMU-check-f	CCCATCCTGATTTATCTGCTTC	Verification of SMUΔ*pplD*
PplD-SMU-check-r	CCATCGTTAGCACTAGCTAGGC	
pplDGBS-check-f	CCATGCTGTTCATGTTATGG	Verification of GBSΔ*pplD*
pplDGBS-check-r	GCATTTGTTGAACGTTGAGG	
H105A_F	CCCAATTTTAATGTATGCTGCTATTCATGTAATGTCC	H105A mutation in PplD, construction of p*pplD*-H105A
H105A_R	GGACATTACATGAATAGCAGCATACATTAAAATTGGG	
D167N_F	CGCCCTTAGACACGCTACCGCTACCAGATGGTAGAGCAAC	D167N mutation in PplD, construction of p*pplD*-D167N and p*pplD*-H105A/D167N
D167N_R	CAATCATACTGTCATTAAATGTTAGCCATACAAC	

[a] Restriction sites are underlined.
[b] Extensions complementary to the antibiotic resistance cassettes or GFP are in bold.

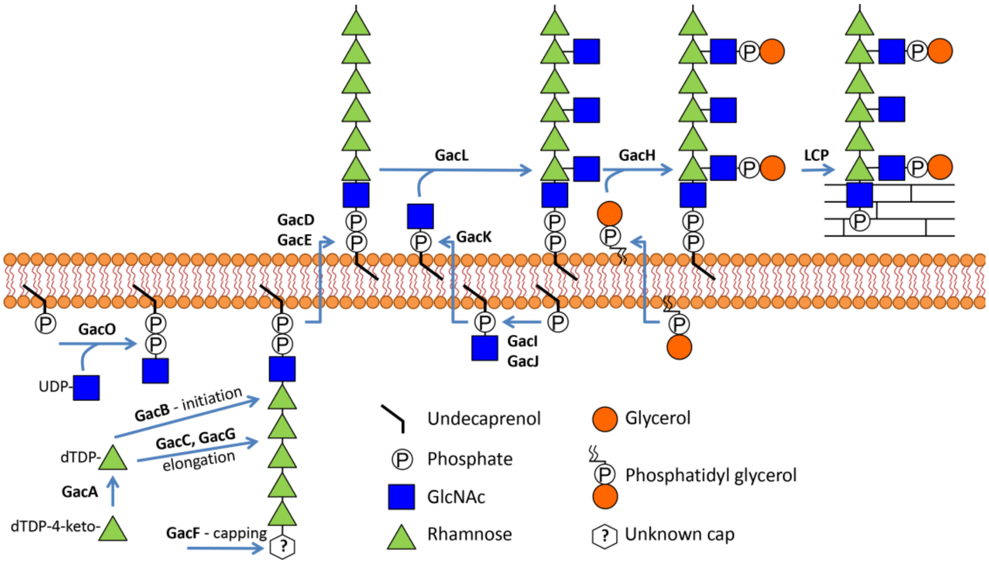

Supplementary Fig. 1. A proposed mechanism of GAC biosynthesis.

GAC biosynthesis is initiated on the inner leaflet of the plasma membrane by GacO which transfers GlcNAc from UDP-GlcNAc to undecaprenyl phosphate (Und-P) producing GlcNAc-P-P-Und [16]. The lipid serves as a membrane-anchored acceptor for GacB-mediated transfer of the first sugar residue, L-Rha, from TDP-β-L-Rha to form Rha-GlcNAc-P-P-Und [25]. The GacC, GacF and GacG glycosyltransferases participate in the elongation step forming the polyrhamnose backbone and capping the structure with unknown sugar residue. Polyrhamnose is transferred to the outer leaflet of the membrane presumably by the GacD/GacE ABC transporter. In the inner leaflet of the membrane, GacI aided by GacJ produces GlcNAc-P-Und [16] which then diffuses across the plasma membrane to the outer leaflet aided by GacK. Subsequently, GacL transfers GlcNAc to polyrhamnose using GlcNAc-P-Und as glycosyl donor [16]. GacH attaches GroP to the GlcNAc side-chains using phosphatidylglycerol as GroP donor [22]. Lastly, protein members of the LytR-CpsA-Psr phosphotransferase (LCP) family presumably attach GAC to peptidoglycan. Several details of this biosynthetic scheme are still speculative. But the overall organization is consistent with other isoprenol-mediated polysaccharide pathways.

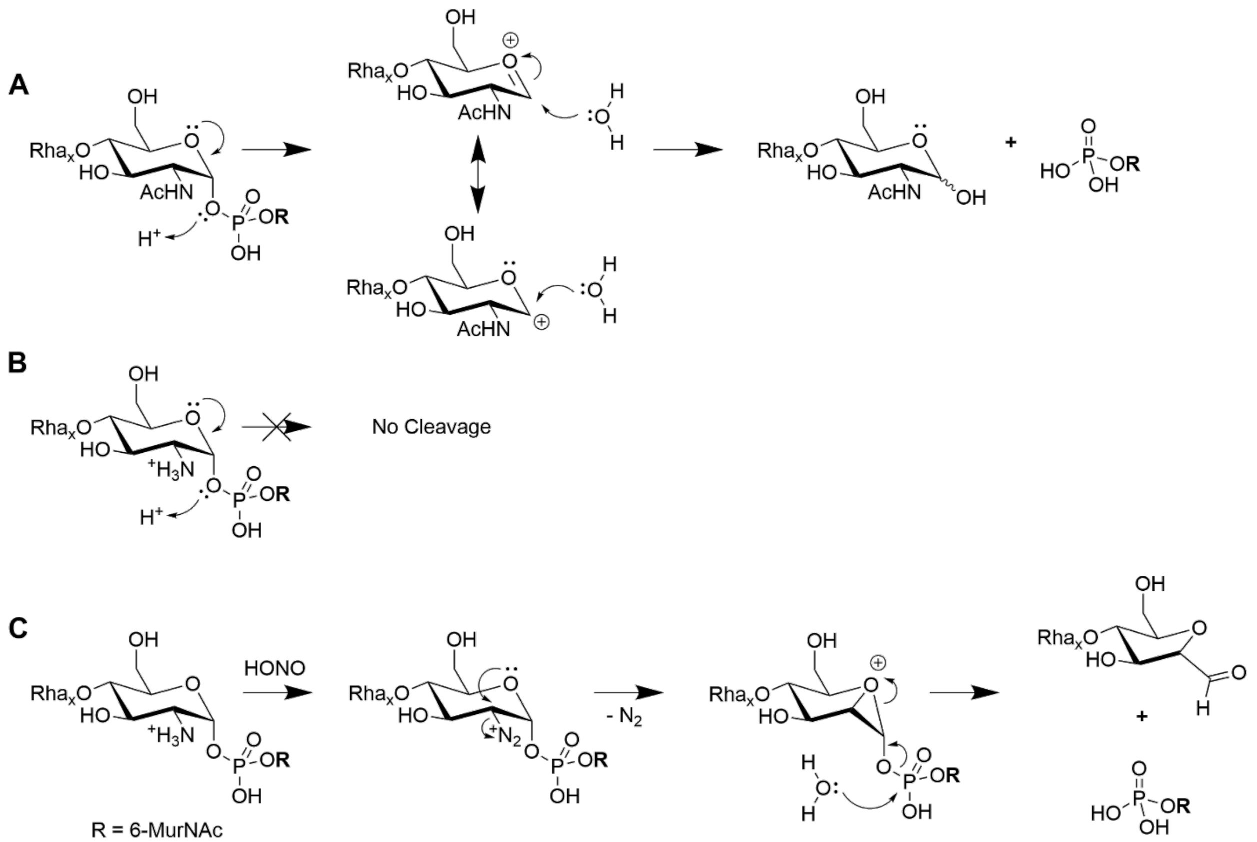

Supplementary Fig. 2. Illustration of reaction mechanisms for hydrolysis of sugar 1-phosphates by 0.02 N HCl and deaminative cleavage of GlcN-1-phosphate by HONO.

(**a**) Acid hydrolysis of GlcNAc 1-phosphate (and hexose 1-phosphates) proceeds efficiently by protonation of the O1-oxygen residue, facilitated by the resonance-stabilized oxonium ion intermediate. (**b**) The positively-charged GlcN-ammonium ion destabilizes the adjacent formation of the oxonium ion intermediate, preventing resonance stabilization of the intermediate. (**c**) Nitrous acid (HONO) forms a diazonium salt at the 2-position of GlcN; intramolecular attack of the GlcN ring oxygen on the diazonium center leads to a bicyclic oxonium intermediate and loss of nitrogen gas. Attack by water on the phosphodiester breaks the C1-O1 bond with concomitant opening of the strained three-membered oxonium ring, resulting in the formation of 2,5-anhydromannose and release of the 6-phosphate MurNAc monoester [26,27].

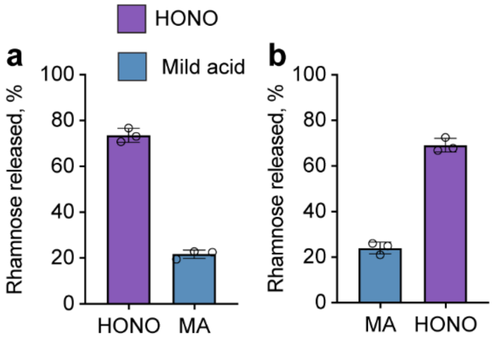

Supplementary Fig. 3. Release of GAC from the GAS cell wall by sequential treatment with either mild acid or HONO.

(**a**) GAC cell wall was subjected to HONO deamination, reisolated, and treated with mild acid. (**b**) GAC cell wall was subjected to mild acid hydrolysis, reisolated and deaminated with nitrous acid. Mild acid hydrolysis and nitrous acid deamination were conducted as described in Methods. The amount of GAC released from cell wall was estimated by the modified anthrone assay and normalized to total GAC content in cell wall. Symbols and error bars represent the mean and S.D. respectively (n = 3 biologically independent replicates). Source data are provided as a Source Data file.

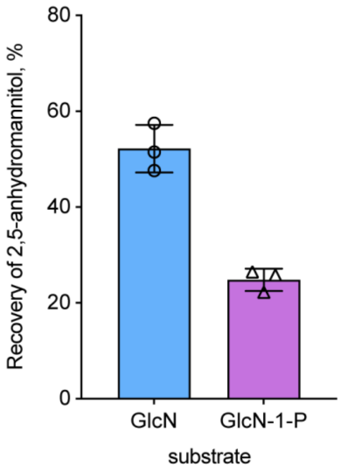

Supplementary Fig. 4. Chemical yield of 2,5-anhydromannitol by HONO deamination of hexosamines.

HONO deamination reactions contained 0.2 N Na acetate, pH 4.5, 1.5 M $NaNO_2$, and 400 nmol of either GlcN (left column) or GlcN 1-phosphate (right column) in a total volume of 0.01 mL. After 90 min at room temperature, the reactions were stopped by the addition of NH_4OH and $NaBH_4$ to a final concentration of 1.5 M and 100 mg/mL, respectively. The reduction reaction was stopped after 2 h by the addition of acetic acid (5 μL) and the reactions were dried repeatedly out of methanol/0.1 % acetic acid under a stream of air to remove borate. The reactions were dissolved in water (344 μL) and an aliquot (5 μL) was taken for analysis. Mannitol (5 nmol, internal standard) was added and the aliquots were dried under air, peracetylated and analyzed by GC/MS as described in Methods for alditol acetate analysis. GlcN-1-phosphate concentration was verified by total phosphate analysis using the malachite green procedure. The amount of 2,5-anhydromannitol formed was calculated as described in Methods using authentic 2,5-anhydromannitol as standard. Symbols and error bars represent the mean and S.D. respectively (n = 3 biologically independent replicates). Source data are provided as a Source Data file.

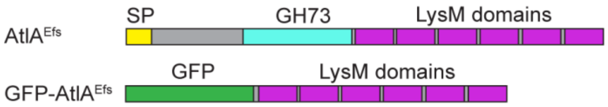

Supplementary Fig. 5. The domain organization of AtlA^{Efs} and the corresponding fluorescent fusion probe GFP-AtlA^{Efs}. GH73 (cyan) denotes the catalytic domain; SP (yellow) indicates signal peptide.

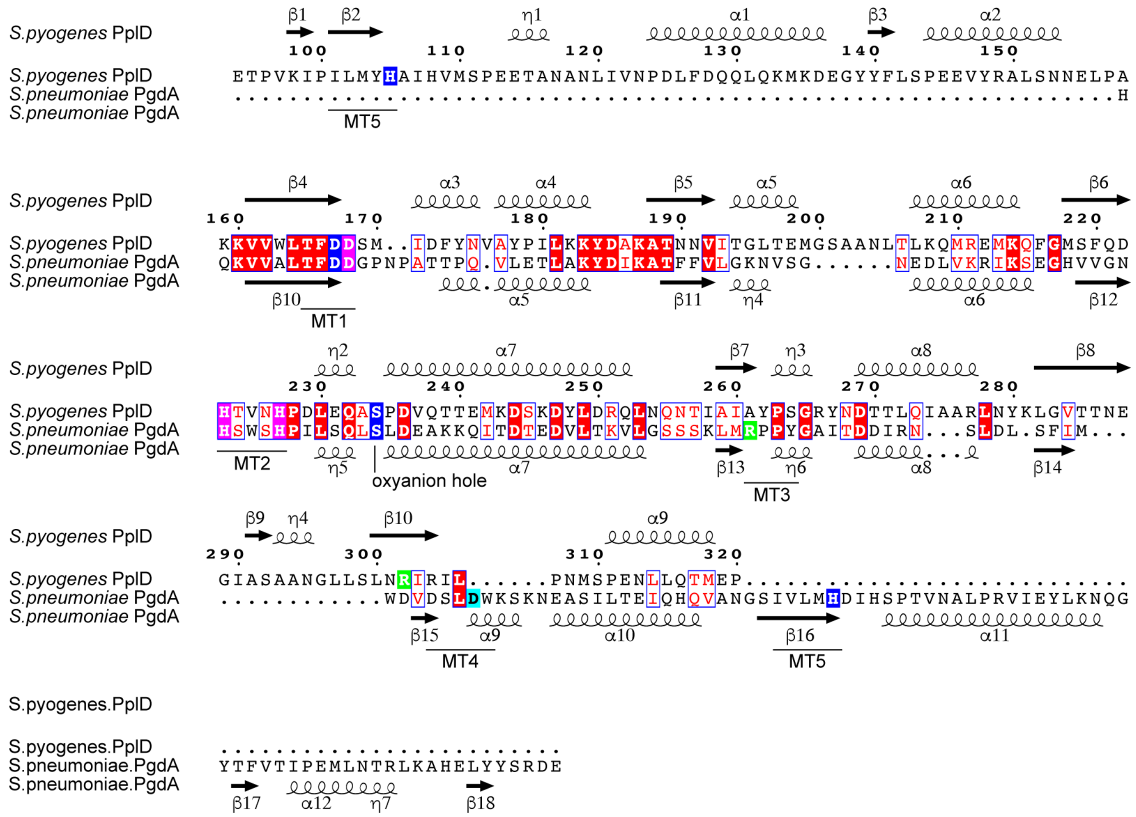

Supplementary Fig. 6. Structure-based sequence alignment of extracellular domains of GAS PplD and *S. pneumoniae* PgdA.

Residues implicated in the catalytic mechanism are highlighted in blue. The secondary structure elements are indicated above (PDB ID 6DQ3) and below (PDB ID 2C1G) [8] alignment.

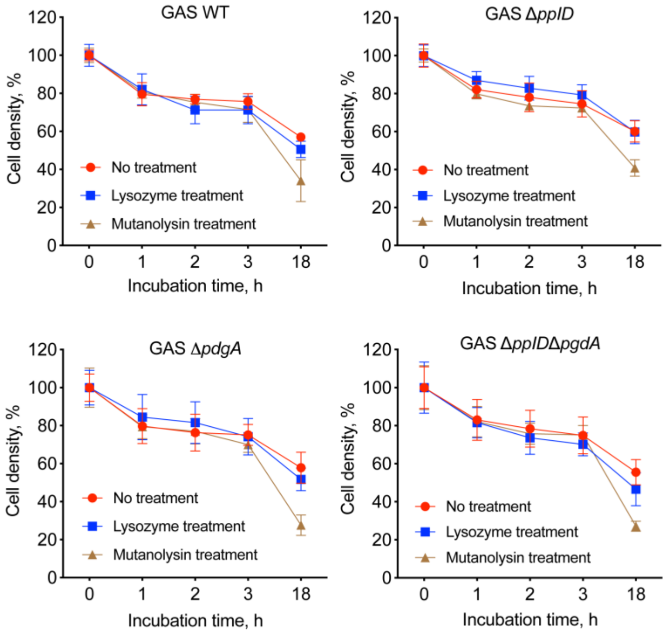

Supplementary Fig. 7. Incubation of GAS WT, GASΔ*pplD*, GASΔ*pgdA*, GASΔ*pplD*Δ*pgdA*, with lysozyme.

Bacterial strains were grown to an OD_{600} of 0.5, centrifuged and resuspended in the same volume of sterile PBS with either 1 mg mL^{-1} lysozyme, 62.5 U/ml mutanolysin (positive control) or no enzyme added (negative control). The bacterial suspension was incubated for 0, 1, 2, 3 and 18 h at 37°C. Lysis was monitored as the decrease in OD_{600}. Results were normalized to the OD_{600} at time zero (OD_{600} of 0.5). Data are mean values ± S.D., n = 3 biologically independent experiments. Source data are provided as a Source Data file.

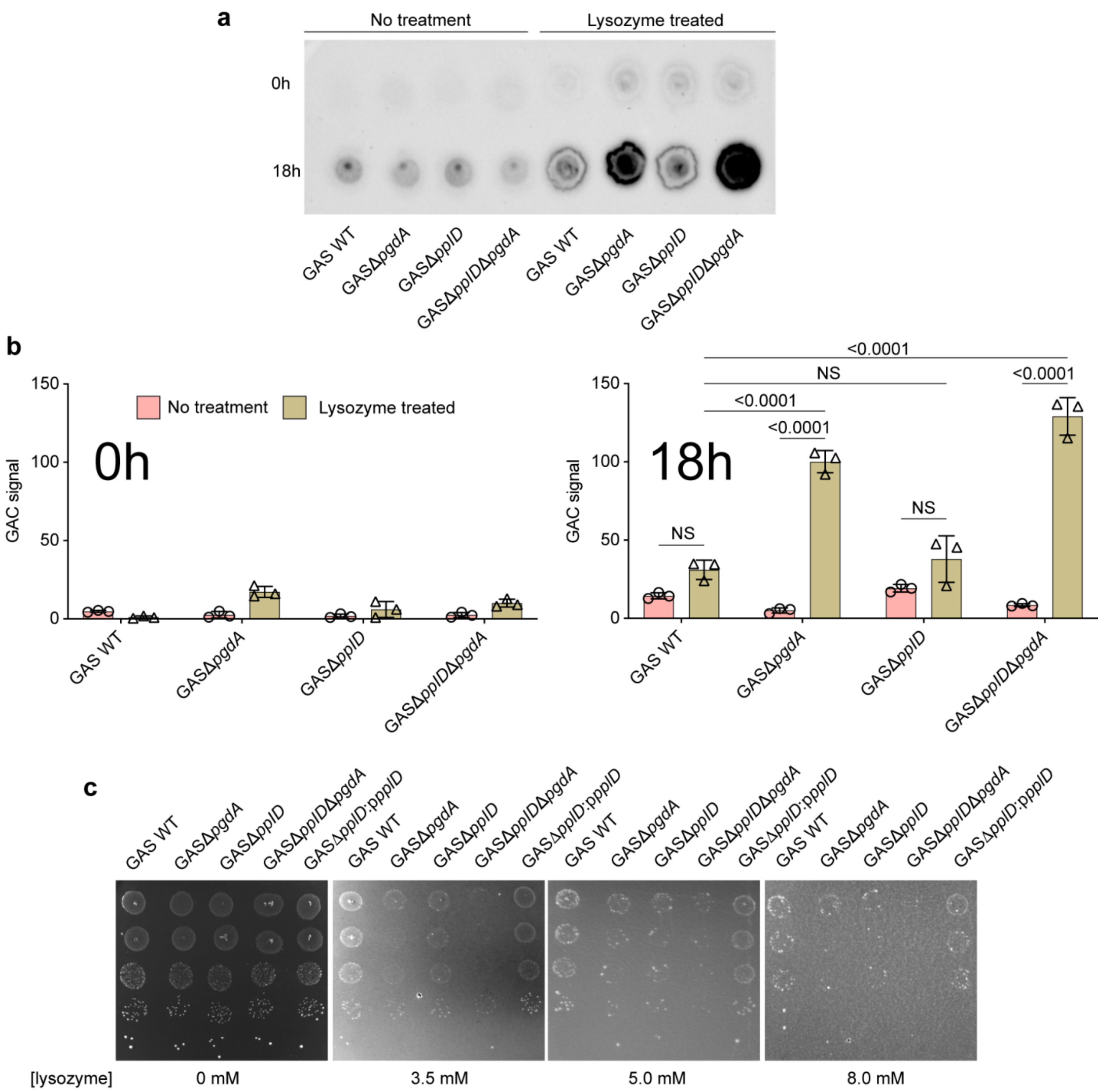

Supplementary Fig. 8. Analysis of lysozyme sensitivity of GAS mutants deficient in *N*-deacetylases PgdA and PplD.

(**a**) Dot-blot analysis for the water-soluble form of GAC released from the WT GAS, GASΔ*pplD*, GASΔ*pgdA* and GASΔ*pplD*Δ*pgdA* cells by lysozyme. GAS samples (OD_{600} of 0.5) incubated for 0 and 18 h with or without lysozyme (1 mg mL^{-1}) were centrifuged (16,000 g, 3 min). The supernatant was concentrated using SpeedVac vacuum concentrator. 5 µl supernatant was spotted on a nitrocellulose membrane. GAC released to the supernatant was detected by anti-GAC antibodies as outlined in Methods. The experiment was performed independently three times and yielded the same results. Representative image from one experiment is shown. (**b**)

Immunoblot of the supernatant (representative image in Supplementary Fig. 8 **a**) collected after 0 (left panel) and 18 h (right panel) of incubation was quantified using ImageJ. Data are mean values ± S.D., n = 3 biologically independent experiments. *P* values were calculated by two-way ANOVA with Tukey's multiple comparisons test. Source data are provided as a Source Data file. (**c**) Lysozyme sensitivity as tested in drop test assay using WT GAS, GASΔ*pplD*, GASΔ*pgdA*, GASΔ*pplD*Δ*pgdA* and GASΔ*pplD*:p*pplD*. Drop test assay experiment was performed at least three times.

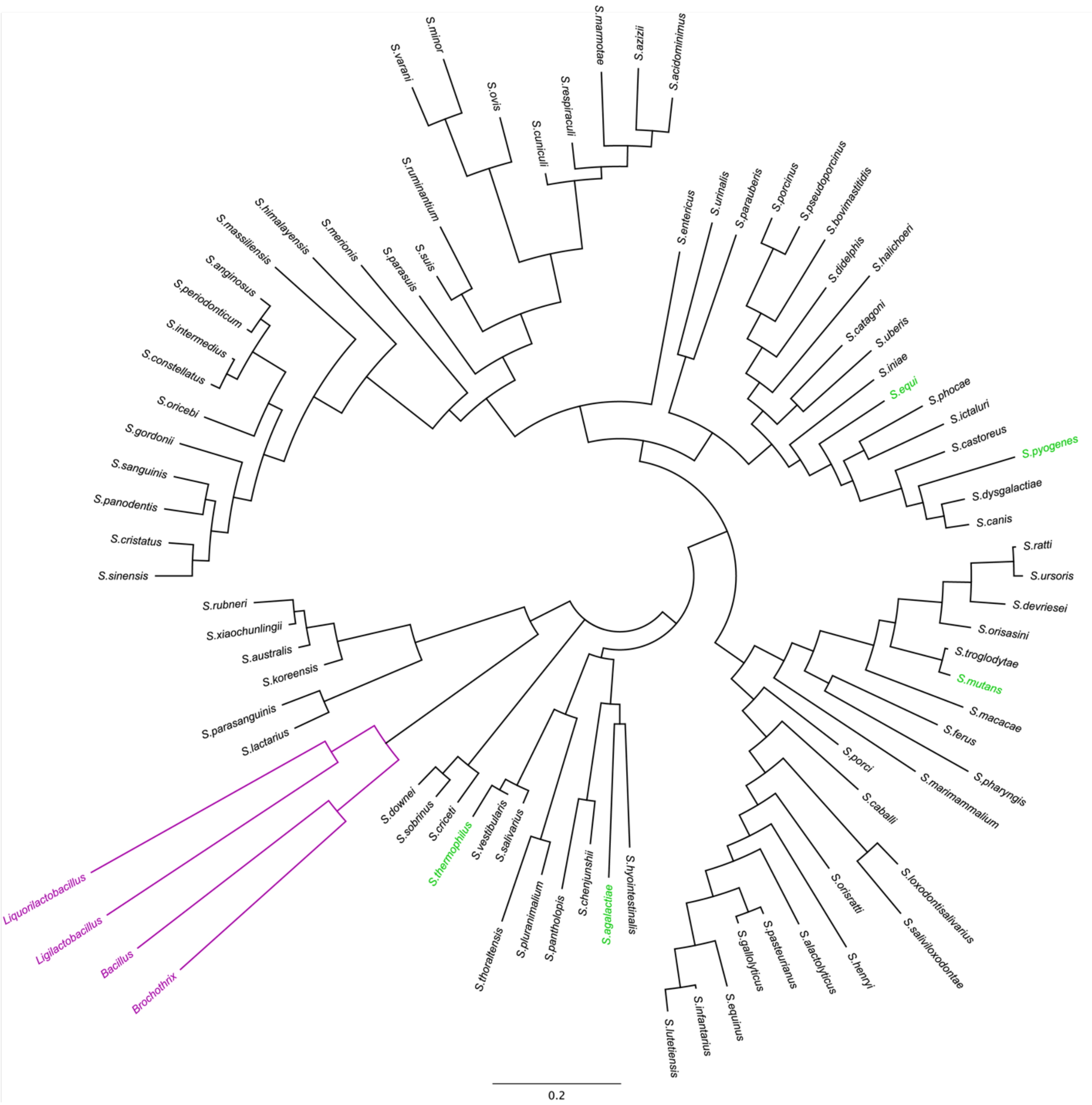

Supplementary Fig. 9. Phylogenetic analysis of PplD homologs.

A phylogenetic tree was generated using the predicted extracellular domains of 86 PplD homologs. The extracellular domains were predicted using the TMHMM server (http://www.cbs.dtu.dk/services/TMHMM/) [28]. There were a total of 234 amino acid positions in the final dataset. The Maximum Likelihood method and JTT matrix-based model [29] were used for evolutionary analysis of PplD homologs. The tree with the highest log likelihood (-16354.06) is shown. Initial tree(s) for the heuristic search were obtained automatically by applying Neighbor-Join and BioNJ algorithms to a matrix of pairwise distances estimated using the JTT model, and then selecting the topology with superior log likelihood value. The tree is drawn to scale, with

branch lengths measured in the number of substitutions per site. Evolutionary analyses were conducted in MEGA X [30,31]. Streptococcal species are shown in black, non-streptococcal species are depicted in purple. *S. pyogenes*, *S. mutans*, *S. agalactiae*, *S. equi* and *S. thermophilus* are shown in green. Sequences of PplD homologs are provided as a Source Data file.

```
                          1        10        20        30        40        50        60
S.agalactiae A909         MAHTPTSHRKPRKRSPWLAIASVFFLLIALIGIFLFFNNRSKQEIKTKTNASSHRKIVTS
S.agalactiae COH1         MAHTPTSHRKPRKRSPWLAIASVFFLLIALIGIFLFFNNRSKQEIKTKTNASSHRKIVTS

                                   70        80        90       100       110       120
S.agalactiae A909         IKKKKWVKQKTPVKIPILMYHAVHVMDPSEAASANLIVAPDIFESHIKRLKKEGYYFLAP
S.agalactiae COH1         IKKKKWVKQKTPVKIPILMYHAVHVMDPSEAASANLIVAPDIFESHIKRLKKEGYYFLAP

                                  130       140       150       160       170       180
S.agalactiae A909         NEAYRALNENALPEKKVIWITFDDGNADFYTKAYPILKKYKVKATNNIITGFVQEGRESN
S.agalactiae COH1         NEAYRALNENALPEKKSHLDYF......................................

                                  190       200       210       220       230       240
S.agalactiae A909         LNVQQMLEMKQNGMSFQGHTVTHPNLSLLTPELQTQEMTLSKLFLDQKLSQDTLAIAYPS
S.agalactiae COH1         ............................................................

                                  250       260       270       280       290
S.agalactiae A909         GRYNPTTLDIASQYYKLGLTTNEGVATKDNGLLSLNRVRILPTTSDDDLIKTINQ
S.agalactiae COH1         .......................................................


                          1        10        20        30        40        50        60
S.thermophilus ASCC 1275  MTSQKKKTSQVKRKKLKLLLLVLNLVLLGLLAVFMLNRPNQSTSNKQQNQTSQSKSTAKW
S.thermophilus LMG 18311  MTSQKKKTSQVKRKN.............................................

                                   70        80        90       100       110       120
S.thermophilus ASCC 1275  KTYDDPVQIPILMYHAVHVMDPSEASNANLIVAPDNFEAQIKAMVDAGYYFLTPEEAYKA
S.thermophilus LMG 18311  ............................................................

                                  130       140       150       160       170       180
S.thermophilus ASCC 1275  FSENVLPAKKVVWLTFDDGNEDFYTIAYPILKKYKAKATNNVITGFVKKGNVGNLTVKQM
S.thermophilus LMG 18311  ............................................................

                                  190       200       210       220       230       240
S.thermophilus ASCC 1275  KEMMAHGMSFQSHTVNHPDLSVTDKATQKDELTNSIDFLEDKLNTKVNTIAYPSGRYNQT
S.thermophilus LMG 18311  ............................................................

                                  250       260       270       280       290
S.thermophilus ASCC 1275  TLGLAKKTYKLGLTTNEGLASANDGLISLNRVRILPTTTAKGLLSKITTDNK
S.thermophilus LMG 18311  ....................................................
```

Supplementary Fig. 10. Frame-shift mutations in *pplD* homologs result in truncated protein products in some streptococci strains.

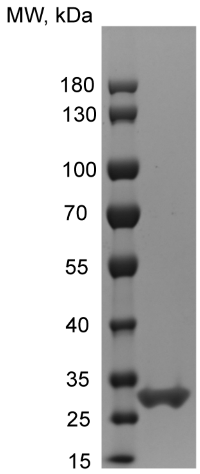

Supplementary Fig. 11. SDS-PAGE of ePplD purified from *E. coli*

ePplD was purified from *E. coli* Rosetta (DE3) cells carrying pCDF-PplD as described in Methods. Protein was separated on 4-12% Bis-Tris Precast gel (GenScript). Representative image from two independent experiments is shown. Uncropped gel image is provided as a Source Data file.

Supplementary references

1 Fadouloglou, V. E. *et al.* Structure determination through homology modelling and torsion-angle simulated annealing: application to a polysaccharide deacetylase from Bacillus cereus. *Acta Crystallogr D Biol Crystallogr* **69**, 276-283, (2013).

2 Arnaouteli, S. *et al.* Two Putative Polysaccharide Deacetylases Are Required for Osmotic Stability and Cell Shape Maintenance in Bacillus anthracis. *J Biol Chem* **290**, 13465-13478, (2015).

3 Andreou, A. *et al.* The putative polysaccharide deacetylase Ba0331: cloning, expression, crystallization and structure determination. *Acta Crystallogr F Struct Biol Commun* **75**, 312-320, (2019).

4 Little, D. J. *et al.* Structural basis for the De-N-acetylation of Poly-beta-1,6-N-acetyl-D-glucosamine in Gram-positive bacteria. *J Biol Chem* **289**, 35907-35917, (2014).

5 Nishiyama, T., Noguchi, H., Yoshida, H., Park, S. Y. & Tame, J. R. The structure of the deacetylase domain of Escherichia coli PgaB, an enzyme required for biofilm formation: a circularly permuted member of the carbohydrate esterase 4 family. *Acta Crystallogr D Biol Crystallogr* **69**, 44-51, (2013).

6 Little, D. J. *et al.* The structure- and metal-dependent activity of Escherichia coli PgaB provides insight into the partial de-N-acetylation of poly-beta-1,6-N-acetyl-D-glucosamine. *J Biol Chem* **287**, 31126-31137, (2012).

7 Little, D. J. *et al.* The protein BpsB is a poly-beta-1,6-N-acetyl-D-glucosamine deacetylase required for biofilm formation in Bordetella bronchiseptica. *J Biol Chem* **290**, 22827-22840, (2015).

8 Blair, D. E., Schuttelkopf, A. W., MacRae, J. I. & van Aalten, D. M. Structure and metal-dependent mechanism of peptidoglycan deacetylase, a streptococcal virulence factor. *Proc Natl Acad Sci U S A* **102**, 15429-15434, (2005).

9 McShan, W. M. *et al.* Genome sequence of a nephritogenic and highly transformable M49 strain of Streptococcus pyogenes. *Journal of bacteriology* **190**, 7773-7785, (2008).

10 Sumby, P. *et al.* Evolutionary origin and emergence of a highly successful clone of serotype M1 group a Streptococcus involved multiple horizontal gene transfer events. *J Infect Dis* **192**, 771-782, (2005).

11 Koga, T., Asakawa, H., Okahashi, N. & Takahashi, I. Effect of subculturing on expression of a cell-surface protein antigen by *Streptococcus mutans*. *Journal of general microbiology* **135**, 3199-3207, (1989).

12 Boschwitz, J. S. & Timoney, J. F. Characterization of the antiphagocytic activity of equine fibrinogen for Streptococcus equi subsp. equi. *Microb Pathog* **17**, 121-129, (1994).

13 Tettelin, H. *et al.* Genome analysis of multiple pathogenic isolates of Streptococcus agalactiae: implications for the microbial "pan-genome". *Proc Natl Acad Sci U S A* **102**, 13950-13955, (2005).

14 Gogos, A., Jimenez, J. C., Chang, J. C., Wilkening, R. V. & Federle, M. J. A Quorum Sensing-Regulated Protein Binds Cell Wall Components and Enhances Lysozyme Resistance in Streptococcus pyogenes. *J Bacteriol* **200**, (2018).

15 Chaffin, D. O., Beres, S. B., Yim, H. H. & Rubens, C. E. The serotype of type Ia and III group B streptococci is determined by the polymerase gene within the polycistronic capsule operon. *J Bacteriol* **182**, 4466-4477, (2000).

16 Rush, J. S. *et al.* The molecular mechanism of N-acetylglucosamine side-chain attachment to the Lancefield group A carbohydrate in *Streptococcus pyogenes*. *J Biol Chem* **292**, 19441-19457, (2017).

17 Mashburn-Warren, L., Morrison, D. A. & Federle, M. J. A novel double-tryptophan peptide pheromone controls competence in Streptococcus spp. via an Rgg regulator. *Mol Microbiol* **78**, 589-606, (2010).
18 Le Breton, Y. & McIver, K. S. Genetic manipulation of *Streptococcus pyogenes* (the Group A Streptococcus, GAS). *Current protocols in microbiology* **30**, Unit 9D 3, (2013).
19 Rajagopal, L., Vo, A., Silvestroni, A. & Rubens, C. E. Regulation of purine biosynthesis by a eukaryotic-type kinase in Streptococcus agalactiae. *Mol Microbiol* **56**, 1329-1346, (2005).
20 Chaffin, D. O. & Rubens, C. E. Blue/white screening of recombinant plasmids in Gram-positive bacteria by interruption of alkaline phosphatase gene (*phoZ*) expression. *Gene* **219**, 91-99, (1998).
21 Jeng, A. *et al.* Molecular genetic analysis of a group A Streptococcus operon encoding serum opacity factor and a novel fibronectin-binding protein, SfbX. *J Bacteriol* **185**, 1208-1217, (2003).
22 Edgar, R. J. *et al.* Discovery of glycerol phosphate modification on streptococcal rhamnose polysaccharides. *Nature chemical biology* **15**, 463-471, (2019).
23 Korotkov, K. V., Delarosa, J. R. & Hol, W. G. J. A dodecameric ring-like structure of the N0 domain of the type II secretin from enterotoxigenic Escherichia coli. *Journal of structural biology* **183**, 354-362, (2013).
24 Korotkov, K. V. & Hol, W. G. J. Structure of the GspK-GspI-GspJ complex from the enterotoxigenic *Escherichia coli* type 2 secretion system. *Nat Struct Mol Biol* **15**, 462-468, (2008).
25 Zorzoli, A. *et al.* Group A, B, C, and G Streptococcus Lancefield antigen biosynthesis is initiated by a conserved alpha-d-GlcNAc-beta-1,4-l-rhamnosyltransferase. *J Biol Chem* **294**, 15237-15256, (2019).
26 Dmitriev, D. A., Knirel, Y. A. & Kochetkov, N. K. Selective cleavage of glycosidic linkages: studies with the model compound benzyl 2-acetamido-2-deoxy-3-*O*-β-D-galactopyranosyl-α-D-glucopyranoside. *Carbohydrate research* **29**, 451-457, (1973).
27 Erbing, C., Lindberg, B. & Svensson, S. Deamination of Methyl 2-Amino-2-deoxy-alpha- and -beta-D-glycopyranosides. *Acta Chemica Scandinavica* **27**, 3699-3704, (1973).
28 Krogh, A., Larsson, B., von Heijne, G. & Sonnhammer, E. L. Predicting transmembrane protein topology with a hidden Markov model: application to complete genomes. *J Mol Biol* **305**, 567-580, (2001).
29 Jones, D. T., Taylor, W. R. & Thornton, J. M. The rapid generation of mutation data matrices from protein sequences. *Computer applications in the biosciences : CABIOS* **8**, 275-282, (1992).
30 Kumar, S., Stecher, G., Li, M., Knyaz, C. & Tamura, K. MEGA X: Molecular Evolutionary Genetics Analysis across Computing Platforms. *Molecular biology and evolution* **35**, 1547-1549, (2018).
31 Stecher, G., Tamura, K. & Kumar, S. Molecular Evolutionary Genetics Analysis (MEGA) for macOS. *Molecular biology and evolution* **37**, 1237-1239, (2020).
