# Supplementary material for: PplD is a de-N-acetylase of the cell wall linkage unit of streptococcal rhamnopolysaccharides
Source: Nat Commun. 2022 Feb 1;13:590. doi: 10.1038/s41467-022-28257-0 (PMC8807736; doi:10.1038/s41467-022-28257-0)
Supplement: Supplementary file 2 — Reporting Summary [file 41467_2022_28257_MOESM2_ESM.pdf]

nature portfolio

Corresponding author(s): Natalia Korotkova

Last updated by author(s): Dec 13, 2021

Reporting Summary

Nature Portfolio wishes to improve the reproducibility of the work that we publish. This form provides structure for consistency and transparency in reporting. For further information on Nature Portfolio policies, see our Editorial Policies and the Editorial Policy Checklist.

Statistics

For all statistical analyses, confirm that the following items are present in the figure legend, table legend, main text, or Methods section.

n/a	Confirmed	
☐	☒	The exact sample size (*n*) for each experimental group/condition, given as a discrete number and unit of measurement
☐	☒	A statement on whether measurements were taken from distinct samples or whether the same sample was measured repeatedly
☐	☒	The statistical test(s) used AND whether they are one- or two-sided<br>*Only common tests should be described solely by name; describe more complex techniques in the Methods section.*
☒	☐	A description of all covariates tested
☐	☒	A description of any assumptions or corrections, such as tests of normality and adjustment for multiple comparisons
☐	☒	A full description of the statistical parameters including central tendency (e.g. means) or other basic estimates (e.g. regression coefficient) AND variation (e.g. standard deviation) or associated estimates of uncertainty (e.g. confidence intervals)
☐	☒	For null hypothesis testing, the test statistic (e.g. *F*, *t*, *r*) with confidence intervals, effect sizes, degrees of freedom and *P* value noted<br>*Give P values as exact values whenever suitable.*
☒	☐	For Bayesian analysis, information on the choice of priors and Markov chain Monte Carlo settings
☒	☐	For hierarchical and complex designs, identification of the appropriate level for tests and full reporting of outcomes
☒	☐	Estimates of effect sizes (e.g. Cohen's *d*, Pearson's *r*), indicating how they were calculated

Our web collection on statistics for biologists contains articles on many of the points above.

Software and code

Policy information about availability of computer code

Data collection	The NMR spectra were recorded on a Bruker AVANCE III 700 MHz equipped with a 5 mm TCI Z-Gradient Cryoprobe (1H/13C/15N) and dual receivers and a Bruker AVANCE II 600 MHz spectrometer equipped with a 5 mm TXI inverse Z-Gradient 1H/D-31P/13C. The spectra were acquired using TopSpin 3.1 software (Bruker BioSpin) for the 600 MHz machine and TopSpin 3.2 (Bruker BioSpin) for the 700 MHz machine. Additional NMR spectra acquired at the Swedish NMR Centre used TopSpin 3.5 pl7 software (Bruker BioSpin) for the 600 MHz machine and TopSpin 3.6.2 (Bruker BioSpin) for the 800 MHz machine. Macromolecular structural data were collected using Blu-Ice 5 (SSRL, beamline 9-2). The data were processed using XDS and XSCALE, version Jan 26, 2018 BUILT=20180126. Fluorescent images were collected using a Leica SP8 equipped with 100X, 1.44 N.A. objective, DIC optics, and “lightning” post-processing. Glycosyl composition analysis was performed using a Thermo ISQ mass spectrometer interfaced with a gas chromatograph, equipped with a 15 m Equity 1701 glass capillary column and helium carrier gas.
Data analysis	Statistical analyses were performed using GraphPad Prism version 9.2.0. The NMR spectra were processed, analyzed and plotted using TopSpin 4.0.1 software (Bruker BioSpin). Macromolecular structural data were analyzed using PHENIX and phenix.refine, version DEV_3139; Phaser version 2.8.2; SHELXD version 2013/2; Parrot version 1.0.4; Buccaneer version 1.6.5; Coot version 0.8.9.1. The Multi-Dimensional Decomposition (MDD-NMR) algorithm was employed to reconstruct the FIDs. Evolutionary analyses were conducted in MEGA X version 10.2.6. Immunoblot data were analyzed using ImageJ version 1.52.

For manuscripts utilizing custom algorithms or software that are central to the research but not yet described in published literature, software must be made available to editors and reviewers. We strongly encourage code deposition in a community repository (e.g. GitHub). See the Nature Portfolio guidelines for submitting code & software for further information.

Data

Policy information about availability of data

All manuscripts must include a data availability statement. This statement should provide the following information, where applicable:

- Accession codes, unique identifiers, or web links for publicly available datasets
- A description of any restrictions on data availability
- For clinical datasets or third party data, please ensure that the statement adheres to our policy

Atomic coordinates and structure factors of PplD crystal structure generated in this study have been deposited in the Protein Data Bank under accession code 6DQ3 [http://dx.doi.org/10.2210/pdb6dq3/pdb]. All data generated during this study are included in the article and Supplementary information files. Source data are provided with this paper.

Field-specific reporting

Please select the one below that is the best fit for your research. If you are not sure, read the appropriate sections before making your selection.

☒ Life sciences ☐ Behavioural & social sciences ☐ Ecological, evolutionary & environmental sciences

For a reference copy of the document with all sections, see nature.com/documents/nr-reporting-summary-flat.pdf

Life sciences study design

All studies must disclose on these points even when the disclosure is negative.

Sample size	No statistical methods were used to determine a sample size, because the sample size was not a factor of the analysis for the experiments. All sample sizes were determined in accordance with published literature relevant to a particular experiment (see for example van Hensbergen, V. P. et al. 2018, PLoS Pathog 14(10):e1007348 and Edgar, R. J. et al. 2019 Nature Chemical Biology 15, 463-471) and they were optimal to generate statistically significant results. In general, the experimental analysis was performed at least in triplicate unless otherwise indicated.
Data exclusions	No data was excluded from analysis.
Replication	All replicates were performed in independent measurements, in different days and similar results were obtained. All experiments were conducted at least three times.
Randomization	Randomization was used to assign 5% crystal diffraction data with the R-free flags during the structure refinement to avoid over-fitting. No randomization was applied to other experiments because the study does not impose a treatment on a group of objects or subjects. Furthermore, selection bias is not relevant to this study because no animals or human subjects were involved, and uncontrollable conditions did not affect the results of the experiments.
Blinding	Blinding was used for microscopy analysis. Investigator was blinded to the group allocation of bacterial cell.

Reporting for specific materials, systems and methods

We require information from authors about some types of materials, experimental systems and methods used in many studies. Here, indicate whether each material, system or method listed is relevant to your study. If you are not sure if a list item applies to your research, read the appropriate section before selecting a response.

Materials & experimental systems

n/a	Involved in the study	
☐	☒	Antibodies
☒	☐	Eukaryotic cell lines
☒	☐	Palaeontology and archaeology
☒	☐	Animals and other organisms
☒	☐	Human research participants
☒	☐	Clinical data
☒	☐	Dual use research of concern

Methods

n/a	Involved in the study	
☒	☐	ChIP-seq
☒	☐	Flow cytometry
☒	☐	MRI-based neuroimaging

Antibodies

Antibodies used	Anti-Streptococcus pyogenes Group A Carbohydrate goat polyclonal antibodies, Abcam, ab9191, lot GR3231184-1; dilution 5,000. Goat anti-rabbit IgG (H+L) conjugated with HRP, ThermoFisher Scientific; 32460; dilution 2,500
Validation	The pAb ab9191 were validated previously by Rush et al. 2017 (J Biol Chem 292, 19441-19457) and Edgar et al. 2019 (Nat Chem Biol

15, 463-471), and has been used extensively in S.pyogenes field. Further validation information is available on the vendor website:
https://www.abcam.com/streptococcus-pyogenes-group-a-carbohydrate-antibody-ab9191.html
Secondary antibodies were validated by respective vendors. Details for general antibody validation or access to certificates of analysis of the respective vendors:
https://www.thermofisher.com/us/en/home/life-science/antibodies/invitrogen-antibody-validation.html
