# Supplementary figures and images for: PplD is a de-N-acetylase of the cell wall linkage unit of streptococcal rhamnopolysaccharides
Source: Nat Commun. 2022 Feb 1;13:590. doi: 10.1038/s41467-022-28257-0 (PMC8807736; doi:10.1038/s41467-022-28257-0)

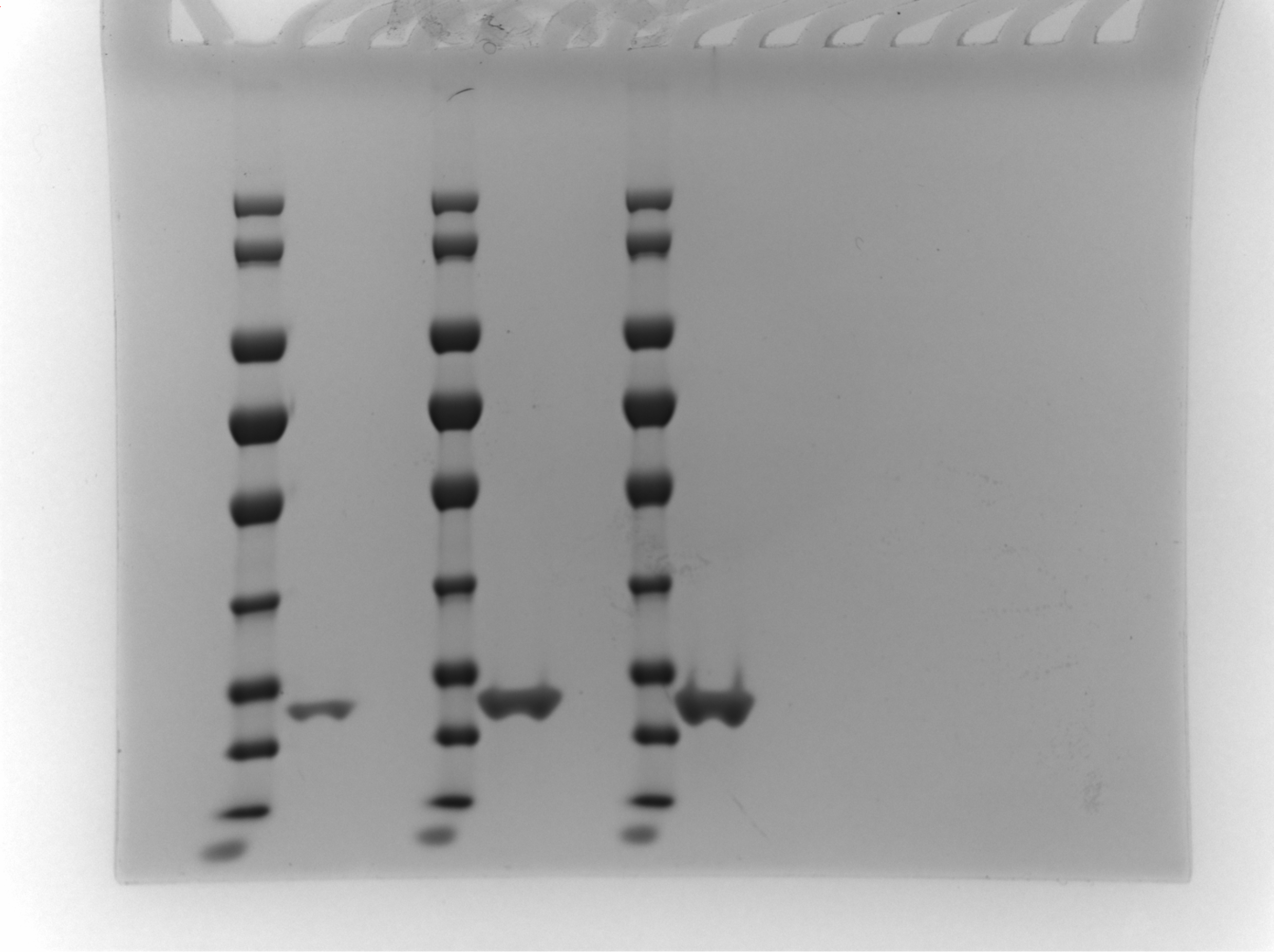

Supplement: Supplementary file 3 — Source data [file 41467_2022_28257_MOESM3_ESM.zip › Rush et al Source data/SupplementaryFigure11.tif]
